# Angioedema and prescribing of omalizumab for chronic urticaria in countries with limited financial resources

**DOI:** 10.1016/j.waojou.2019.100079

**Published:** 2019-10-23

**Authors:** Todor A. Popov, Martin K. Church, George Christoff, Marcus Maurer

**Affiliations:** a University Hospital "Sv. Ivan Rilski", Sofia, Bulgaria; b Charité – University Medical Center, Berlin, Germany; c Faculty of Public Health, Medical University Sofia, Sofia, Bulgaria

Dear Editor,

Urticaria comprises a spectrum of conditions characterized by the appearance of itchy wheals. If the symptoms recur for longer than 6 weeks (average duration is 5–7 years), the condition falls within the definition of chronic urticaria.^1^ In more than half of the cases of chronic urticaria (CU), the wheals are accompanied by swelling of the deeper cutaneous and subcutaneous tissues referred to as angioedema, which can persist for several days.^2^ Angioedema often develops in the face, lips and oral cavity. In around 10% of affected patients, angioedema occurs in the absence wheals. In this case, hereditary angioedema and other forms of bradykinin mediated angioedema have to be ruled out, as the two conditions have very different pathogenic mechanisms and associated risks.^3^ When determining the diagnosis and management of CU, wheals and angioedema are generally viewed together: disease severity is established on the basis of subjective and objective indicators, treatment starts with the licensed doses of H_1_-antihistamines, which may be subsequently increased up to 4 times.^4^

Patients with severe CU, who do not respond to off-label high doses of second generation H_1_-antihistamines pose serious problems to the treating physicians.^1^ Omalizumab (anti-IgE) has been proven to substantially increase the success rate of treatment in such antihistamine non-responsive cases.^1^^,^^5^ Omalizumab sequesters IgE, the classical antibody associated with allergic diseases and asthma. In so doing so, omalizumab cuts short multiple mast cell activation mechanisms, suppresses the release of mediators, including histamine, and prevents pathological symptoms.^6^ Omalizumab, thus, represents a treatment option with societal benefit with the cost of the product itself being counterbalanced by the effect on indirect (productivity) costs.^7^

Omalizumab is approved for the treatment of antihistamine-resistant chronic spontaneous urticaria (CSU) in over 80 countries around the world and is licensed by the European Medicines Agency of the European Union and the Food and Drug Administration of the USA. However, if reimbursement is not provided by the healthcare systems in the countries where it is licensed, its accessibility for treatment is precluded by its relatively high cost. An argument of the health authorities to keep it off reimbursement lists is that CU is not a fatal disease and the regulators in countries with limited financial resources do not attach to omalizumab enough weight to override the reimbursement threshold. However, the presence of angioedema imparts to CU a higher level of importance, as the general public perceives the condition as life threatening.^1^

The very first episode of facial edema and sense of swelling in the oral cavity and the throat triggers an existential fear of suffocation in patients with CU. The dramatically altered psycho-emotional state of those affected is conveyed to the emergency medical staff: high doses of parenteral corticosteroids are applied and often hospitalization is proposed. The patients become convinced that they have had a close encounter with death, and the horror of future similar episodes is seeded in them. In most cases, systemic corticosteroids are prescribed,^8^ and attempts to discontinue them are usually followed by the resurgence of CU/angioedema symptoms, thereby initiating a vicious circle of chronicity. Based on our clinical experience, the real risk for patients with chronic urticaria associated angioedema is addiction to systemic corticosteroids and the ensuing side effects that can trigger other chronic diseases (e.g. osteoporosis, diabetes, arterial hypertension, ulcer, obesity) and disturb the overall hormonal balance (particularly unwanted for female patients).^9^ As in asthma,^10^ the shadow cost of oral corticosteroid-related adverse events is likely to have a significant unwanted economic impact on society and health insurance systems (Fig. 1).

**Fig. 1:**
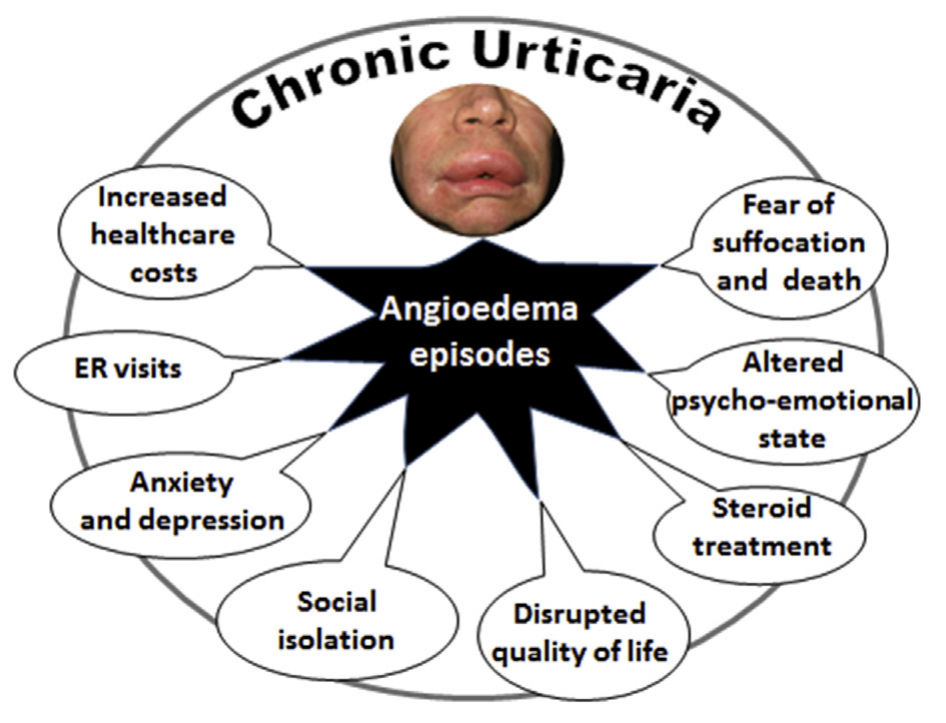
Infographic of the shortcomings following the first episode of angioedema in patients with chronic urticaria. ER = Emergency Room

While omalizumab, like other biologics, may appear relatively expensive, the arguments for its reimbursement for the treatment of CU are several. First, angioedema occurs frequently in patients with CU, one report indicating an incidence of up to 71%.^11^ Second, angioedema is commonly underdiagnosed,^12^ and it may happen at any time during the course of the disease. Third, in CU patients with angioedema, angioedema episodes are frequent. In a recent study on 91 CU patients with angioedema, 60% of patients had angioedema weekly.^13^ Fourth, 9 of 10 patients experience angioedema of the lips and more than half report angioedema of the tongue, the mouth and upper airways.^13^ Fifth, in 45% of patients, the duration of angioedema is more than 24 hours. Sixth, in a study of 665 patients with CU, the severity of angioedema was assessed as moderate or severe in 78% of patients.^14^ Seventh, omalizumab has been shown to decrease systemic corticosteroid use in most CU cases in 1 retrospective study^15^ and to have steroid sparing effect in a case report^16^: thus, it may reduce the possibility of corticosteroid-related adverse events. Finally, and perhaps most importantly, angioedema markedly impairs quality of life, even in patients with low wheal scores, and often leads to social isolation.^11^^,^^17^ Although omalizumab provides an effective treatment for CU patients with angioedema, it is all too often not available to them because of the failure of health authorities to reimburse it.^1^^,^^13^^,^^18^^,^^19^

## Consent for publication

All authors have reviewed and approved of the final manuscript.

## Ethics approval

No actual work with human subjects or animals had been conducted in relation to this letter-to-editor.

## Declaration of competing interest

None of the authors has any competing interests.
